# Dimensionality, Sensitivity and Specificity of Different Versions of the Shirom‐Melamed Burnout Questionnaire/Measure in Clinical and Non‐Clinical Populations

**DOI:** 10.1002/smi.70001

**Published:** 2025-01-20

**Authors:** Anna E. Sundström, Maria Nordin, Steven Nordin, Anna Stigsdotter Neely, Hanna Malmberg Gavelin

**Affiliations:** ^1^ Department of Psychology Umeå University Umeå Sweden; ^2^ Department of Health Education and Technology Luleå University of Technology Lulea Sweden; ^3^ Department of Social and Psychological Studies Karlstad University Karlstad Sweden; ^4^ Department of Public Health and Clinical Medicine Section of Sustainable Health Umeå University Umeå Sweden

**Keywords:** burnout, clinical, construct validity, SMBM, SMBQ, stress‐related illness

## Abstract

The Shirom‐Melamed Burnout Questionnaire/Measure (SMBQ/SMBM) is a self‐report instrument frequently used for assessing degree of burnout and screening for stress‐related exhaustion disorder. The aim of the present study was three‐fold. First, to examine reliability and construct validity of different versions of SMBM with 6–22 items in a clinical context. Second, to examine the criterion validity by assessing sensitivity and specificity and determining clinical cut‐offs for these versions of the SMBM, and third to examine the prevalence of burnout in a general population and primary care sample using the proposed cut‐offs. Two Swedish samples were used for the first two purposes: a clinical sample of patients diagnosed with exhaustion disorder (*n* = 149), and a matched sample of healthy controls (*n* = 60). For the third purpose a sample from the general population (*n* = 3406), and a primary care clinical sample (*n* = 326) was used. The modified versions of the SMBM showed good internal consistency, construct validity, dimensionality and model fit on the clinical exhaustion disorder sample, as well as configural measurement invariance across clinical and non‐clinical samples. The sensitivity (94.6%–95.3%) and specificity (93.3%–95.0%) in identifying cases with exhaustion disorder based on the cut‐off of 4.0 for the 19‐, 16‐ and 11‐items versions, and on the cut‐off of 3.75 for the 6‐item version was high. The prevalence of burnout was 81.2% in the primary care sample and 16.6% in the general population sample. The findings indicate that the SMBM is a useful instrument for screening for exhaustion disorder and burnout.

## 1 Introduction

Stress‐related illness, such as burnout, in the general population is a major concern in many countries (Salari et al. 2020), and associated with substantial societal costs due to increased health care consumption, sickness absence, loss in productivity and employee turnover (Edú‐Valsania, Laguía, and Moriano 2022; Hassard et al. 2018). In Sweden, mental ill‐health is the primary cause for sick‐leave, and stress‐related disorders constitute the diagnostic category for which sick‐leave rates are increasing the most (The Swedish Social Insurance Agency 2020).

Burnout is a mental condition characterised by prolonged feelings of physical fatigue, emotional exhaustion and cognitive weariness, that results from continuous stress exposure (Melamed et al. 2006). Traditionally, burnout has been associated with work‐related stress and not regarded as a clinical condition. In the 11th revision of the International Classification of Diseases (ICD‐11), burnout is included as an occupational phenomenon, defined as a syndrome resulting from chronic workplace stress that has not been successfully managed (WHO 2019). In the Swedish version of ICD‐10 however, a clinical level of burnout is recognised as a mental health disorder termed stress‐related exhaustion disorder (diagnostic code 43.8A, see Table A1 for diagnostic criteria) (The National Board of Health and Welfare 2003). The clinical diagnosis exhaustion disorder is used in Swedish health care practice for individuals who have fallen ill due to long‐term psychosocial stress exposure (Grossi et al. 2015). Exhaustion disorder is characterised by physical and mental exhaustion of minimum of 2 weeks duration and the diagnostic criteria stipulate that these symptoms have developed in response to identifiable stressors, present for at least 6 months. Exhaustion disorder is associated with multifaceted, long‐lasting symptoms such as cognitive difficulties, disturbed sleep, sensitivity to stress as well as somatic complaints (Glise et al. 2012, 2020; Lindsäter et al. 2023) and long sick‐leave periods (The Swedish Social Insurance Agency 2020). There are equivalents to exhaustion disorder in the international literature such as clinical burnout and stress‐related exhaustion (Wallensten et al. 2019). The definition of clinical burnout is usually based on the criteria for work‐related neuroasthenia in ICD‐10 (van Dame 2021) which comprises complaints about fatigue after mental effort or complaints of bodily weakness and exhaustion after minimal effort as well as additional symptoms of insomnia, cognitive deficits, and somatic symptoms.

Burnout measures are often used by clinicians in the diagnostic process to measure illness severity and outcomes of treatment in exhaustion disorder (Lindsäter et al. 2022; van de Leur et al. 2023). One widely used measure in both research and clinical practice is the Shirom‐Melamed Burnout Questionnaire/Measure (SMBQ/M). The SMBQ/M is based on the definition that burnout is a long‐term affective state consisting of emotional exhaustion, physical fatigue and cognitive weariness symptoms that arises from the depletion of energetic resources due to cumulative exposure to chronic stressors both at and off work (Melamed et al. 2006). Thus, the contents of the SMBQ/M cover aspects of the diagnostic criteria for exhaustion disorder concerning physical and emotional exhaustion as well as cognitive impairments, while diagnostic criteria related to sleep disturbance, physical symptoms and increased sensitivity to stress are not covered by the instrument.

There are different versions of the SMBQ/M encompassing different subscales and number of items, and as noted by Almén and Jansson (2021), there are ambiguities regarding what constitutes the SMBQ and SMBM respectively. Therefore, in accordance with Shirom and Melamed (2006) as well as Almén and Jansson (2021) we chose to use the acronym SMBM for all versions of the SMBQ/SMBM. The 22‐item SMBM consists of four subscales: Emotional and Physiological Exhaustion (EPE), Cognitive Weariness (CWE), Listlessness (LIS) and Tension (TES) (Lundgren‐Nilsson et al. 2012). The EPE and CWE subscales capture the core aspects of the burnout construct, that is, feelings of emotional, physical, and cognitive exhaustion (Melamed, Kushnir, and Shirom 1992; Shirom and Melamed 2006). In contrast, the TES subscale is intended to capture the feelings of tension that arise from unsuccessful coping during the early stages of the burnout process, whereas the LIS subscale aims to capture feelings of withdrawal and apathy in the later stages of burnout (Melamed, Kushnir, and Shirom 1992). There is also a subscale of emotional exhaustion in relation to customers that has been used in some studies (Melamed, Kushnir, and Shirom 1992). Since this subscale encompasses items that apply only to working populations it was not included in the present study. The brief versions of the SMBM, referred to as the SMBM‐11 and SMBM‐6 in the present study, cover the core aspects of the burnout construct; physical fatigue, emotional exhaustion and cognitive weariness (Almén and Jansson 2021; Qiao and Schaufeli 2011).

The SMBM has been translated from English into several languages, including for example Swedish, French, German, Polish, Russian and Spanish, and its measurement properties (Mokkink et al. 2010) have been examined in several studies (Shoman, Hostettler, and Canu 2023). In a recent review, Shoman, Hostettler, and Canu. (2023) concluded that SMBM shows good evidence for construct validity and internal consistency, while content, structural and criterion validity were rated as insufficient. For example, Schilling et al. (2019) reported that the SMBM demonstrated adequate psychometric properties as well as construct validity on German working population samples and that measurement invariance across gender was observed. Gerber et al. (2018) found that the SMBM has excellent psychometric properties and acceptable convergent and discriminant validity in adolescent samples, and thus is a suitable screening instrument for burnout also among adolescents. In a Swedish context, the reliability and construct validity of both the SMBM‐22 and the modified shorter versions have recently been demonstrated (Almén and Jansson 2021, 2023; Sundström et al. 2023). The modified versions include a 4‐factor 19‐item version, which, in a normal population sample, demonstrated better psychometric properties compared to the 22‐item version (Almén and Jansson 2021; Sundström et al. 2023). Moreover, based on the work by Lundgren‐Nilsson et al. (2012) an 18‐item 3‐factor SMBM has been proposed to assess the EPE, CWE and LIS subscales (TES subscale excluded), and this version was further modified to a 16‐item 3 factor version by Almén and Jansson (2021). Furthermore, two 2‐factor versions; SMBM‐11 and SMBM‐6, covering the core dimensions of the burnout construct (EPE and CWE) have demonstrated good psychometric properties in three different Swedish community samples (Almén and Jansson 2021, 2023; Sundström et al. 2023). These versions have been suggested to be useful when brief measures focusing on the core components of burnout are needed, for example, to reduce participant burden or when conducting repeated assessments (Almén and Jansson 2021; Sundström et al. 2023).

The SMBM uses clinical cut‐off scores to distinguish between those experiencing normal levels of stress and those at risk of exhaustion disorder or clinical burnout. To date, different cut‐offs for the Swedish SMBM‐22 have been used in the scientific literature to identify clinical cases or at‐risk individuals. For example, in a study on the physiological correlates of burnout among women, Grossi et al. (2003) used quartile splits to define a high burnout group as those scoring 3.75 and above; this cut‐off has subsequently been used in other studies (e.g. Glise et al. 2010; Wiegner et al. 2015). More recently, Glise, Ahlborg, and Jonsdottir (2012, 2014) used a cut‐off of 4.0 for clinical burnout, which was determined based on clinical experience and expert consensus. Although these cut‐offs have been adopted into both research (e.g., Norlund, Reuterwall, Höög, Lindahl, Janlert and Birgander 2010; Soares, Grossi, and Sundin 2007) and clinical settings, they were not determined based on clinical diagnosis. Conversely, Lundgren‐Nilsson et al. (2012) used data from both a clinical sample consisting of patients diagnosed with exhaustion disorder, and a general working population, suggesting a cut‐off of 4.4 to identify clinical cases of burnout in the SMBM‐22 as well as the SMBM‐18. In their study, the suggested cut‐off score would place 83.4% of the clinical sample above the cut‐off (sensitivity). However, the specificity could not be estimated as the sample from the general population not only included healthy controls.

As referred to above, two recently published studies on the construct validity of the SMBM have suggested that the SMBM‐19 has superior psychometric properties to that of the SMBM‐22. Moreover, two shorter versions SMBM‐11 and SMBM‐6, have been suggested to assess the core of the burnout construct that is, exhaustion and cognitive weariness (Almén and Jansson 2021; Sundström et al. 2023). There are, however, very low evidence of criterion validity for the SMBM in general (Shoman, Hostettler, and Canu 2023) and no clinical cut‐offs has been established for these brief versions yet. Given that brief measures can be very useful in clinical as well as research settings, there is an urgent need of establishing clinical cut‐offs for these versions. In addition, there is a need to examine the construct validity and internal consistency of these different versions of the SMBM on a clinical sample, as previous psychometric studies mainly have used community samples.

## 2 Aim

The aim of the present study was three‐fold. First, to examine the reliability and construct validity of the proposed versions of the SMBM by assessing the dimensionality and measurement invariance across clinical and non‐clinical samples, as well as inter‐correlations between versions of SMBM and mean score differences between clinical and non‐clinical samples. Second, to examine the criterion‐related validity of the proposed versions of the SMBM by assessing specificity and sensitivity and determining clinical cut‐offs. Third, to examine the prevalence of burnout in a general population and a primary care sample using these proposed cut‐offs.

## 3 Method

### 3.1 Participants and Procedure

In the present study four samples were employed. In order to meet the first and second aim a clinical exhaustion disorder sample and a matched sample of healthy controls were used. To fulfil the third aim a large sample from the Swedish general population as well a sample of patients with stress‐related ill‐health from primary care were used. These samples are described in more detail below.

#### 3.1.1 Clinical Exhaustion Disorder Sample and Healthy Controls

The patients with clinical diagnosis of exhaustion disorder included in the study were part of the Rehabilitation for Improved Cognition (RECO) study, a randomised clinical trial conducted at the Stress Rehabilitation Clinic at the University Hospital of Umeå, Sweden (ClinicalTrials.gov: NCT03073772). The results and procedure of this trial have previously been described in detail (Gavelin et al. 2018). Briefly, the RECO study investigated the effects of cognitive and aerobic training, offered as additional treatments to a 24‐week multimodal stress rehabilitation programme. Data from the baseline assessment, before patients started the stress rehabilitation programme, were used for the current study. Patients were recruited from April 2010 to June 2013. Inclusion criteria for the patient group were (1) diagnosis of exhaustion disorder according to the diagnostic criteria included in the Swedish version of the ICD‐10 (code F43.8A), confirmed by a physician; (2) aged 18–60 years; (3) currently employed; (4) considered by a physician and a psychologist to be suitable for group‐based multimodal stress rehabilitation; (5) no known abuse of alcohol or drugs; (6) not in need of other more urgent treatment; and (7) not participating in other interventional study. Of the 161 patients who participated in the baseline assessment, 149 completed the SMBM‐22 and were included in the present study. A matched healthy control group were recruited in the spring of 2016 through advertisement in a local newspaper (see Nelson et al. 2021). Out of 109 respondents, the first 60 matching the patient group on the variables age and sex were invited to participate. An initial telephone interview was conducted to exclude those with self‐reported history of stress‐related exhaustion or other medical and/or psychological conditions known to affect cognition. Among the control group participants, a majority were women, married or cohabitant and had a university level education.

In the clinical exhaustion disorder sample, most of the participants were women, and 87.6% were partly or fully on sick leave. There were 59% of the participants that were on sick leave ≥ 75%. The majority of the participants in the clinical exhaustion disorder sample (56%) had been on sick leave for six months or less before starting the multimodal stress rehabilitation programme, while 16% had no sick leave prior to starting the treatment. Smaller proportions had been on sick leave for 7–12 months (14%), 12–24 months (6%), or more than 24 months (8%). The characteristics of the clinical exhaustion disorder sample and healthy control sample are shown in Table 1. No differences in sex or age distribution were found between these samples. The study was conducted in accordance with the Helsinki Declaration and approved by the Umeå Regional Ethical Review Board (Dnr 2010‐53‐31, 2015‐475‐32M). Participation in the study was strictly voluntary and all participants gave their informed consent.

**TABLE 1:** Sample characteristics.

Variable	Clinical exhaustion disorder sample (*n* = 149)	Healthy control sample (*n* = 60)	General population sample (*n* = 3406)	Clinical primary health‐care sample (*n* = 326)
Age, mean (SD)	44 (8.6)	44 (6.6)	51 (16.9)	40 (13.3)
Min–max	22–60	35–56	18–79	17–83
Gender, *n* (%)				
Women	122 (82%)	48 (80%)	1898 (56%)	264 (70%)
Men	27 (18%)	12 (20%)	1508 (44%)	106 (28%)
Education, *n* (%)				
Elementary school	9 (6%)	2 (3%)	(24.5%)	16 (4.3%)
High school	49 (33%)	22 (37%)	(33.8%)	103 (27.5%)
University	91 (62%)	36 (60%)	(41.8%)	236 (63.1%)
Married/cohabitant, *n* (%)	115 (77%)	41 (68%)	2480 (72.8%)	275 (73.3%)
Sick‐leave, *n* (%)				
100%	71 (48%)	—	—	29 (9.0%)
75%	17 (11%)	—	—	14 (4.3%)
50%	28 (19%)	—	—	21 (6.5%)
25%	16 (11%)	—	—	1 (2.2%)
0%	17 (11%)	—	—	253 (78.1%)

#### 3.1.2 Sample From a Swedish General Population

Using the clinical cut‐offs established in the present study, the prevalence of exhaustion in the Swedish general population were examined using a sample of adults (18–79 years) from the Västerbotten Environmental Health Study (VEHS), in which SMBM‐22 was used for assessing burnout. The participants were recruited from the population in the county of Västerbotten in Northern Sweden, which has an age and gender distribution similar to that of Sweden in general (Palmquist et al. 2014). Data collection was carried out during 2010 by using questionnaires sent to a random sample (*N* = 8520), stratified for age and sex. 3406 participants responded to the questionnaire (corresponding to a response rate of 40%). Sample characteristics are shown in Table 1. The study was conducted in accordance with the Helsinki Declaration and approved by the Umeå Regional Ethics Board (Dnr 09–171 M) and the Swedish Ethical Review Authority (Dnr 2022‐05265‐02). Participation in the study was strictly voluntary and all participants gave their informed consent.

#### 3.1.3 Clinical Sample of Patients From Primary Care

The clinical cut‐offs established in the present study were also applied on a clinical sample from the primary health care in northern Sweden. The participants were primary care patients experiencing stress‐related ill‐health or sleep disturbance, taking part in short psycho‐educative group treatments for stress and sleep given by trainee psychologists under supervision of clinical psychologists. Data was collected during 2020–2023 through a questionnaire administered before treatment. In total 393 participants gave their written consent to participate in the study, of those 326 had completed the SMBM‐22 and were included in the present study. Participation in the study was strictly voluntary and all participants gave their informed consent. The study was conducted in accordance with the Helsinki Declaration and approved by the Swedish Ethical Review Authority (Dnr 2020‐00168).

### 3.2 Instruments/Materials

#### 3.2.1 Shirom‐Melamed Burnout Measure

The SMBM‐22 was used to assess degree of burnout. Each item in the subscales EPE, CWE, LIS and TES, are rated on a 7‐point scale (1 = ‘almost never’, 7 = ‘almost always’). The EPE subscale consists of eight items, examples of which include ‘I feel tyred’ and ‘I feel burned out’. The CWE subscale comprises six items, such as ‘I have difficulties concentrating’ and ‘My thinking process is slow’. Four items measure LIS, including ‘I feel sleepy’ and ‘I feel active’ (with reversed scoring). The TES subscale comprises four items, including ‘I am tense’ and ‘I feel restless’. The mean score across all items were used, with high score reflecting a high level of burnout. Item content is displayed in Table 2.

**TABLE 2:** The items used in the different versions of the SMBM and their associated factor loadings in the clinical exhaustion disorder sample.

Item	Scale version					
	Subscale	4‐Factor SMBM‐22	4‐Factor SMBM‐19	3‐Factor SMBM‐16	2‐Factor SMBM‐11	2‐Factor SMBM‐6
1. I feel tyred (Jag känner mig trött)	EPE	0.798	0.720	0.728	0.700	—
2. I feel refreshed (reversed) (Jag känner mig pigg)	EPE	0.791	—	—	—	—
3. I feel physically exhausted (Jag känner mig fysiskt utmattad)	EPE	0.606	0.614	0.610	0.641	0.589
4. I feel “fed up” (Jag känner att jag har fått nog)	EPE	0.455	0.511	0.498	0.510	0.506
5. I feel full of vitality (reversed) (Jag känner mig full av energi)	LIS	0.624	0.676	0.673	—	—
6. My batteries are dead (Mina batterier är uttömda)	EPE	0.765	0.797	0.803	0.811	—
7. I feel alert (reversed) (Jag känner mig alert)	LIS	0.700	0.747	0.747	—	—
8. I feel burned out (Jag känner mig utbränd)	EPE	0.706	0.766	0.762	0.768	0.804
9. I feel mentally fatigued (Jag känner mig mentalt trött)	EPE	0.587	0.605	0.607	—	—
10. I feel no energy for going to work in the morning (Jag känner att jag inte orkar gå till arbetet på morgonen)	EPE	0.603	0.645	0.641	0.645	—
11. I feel active (reversed) (Jag känner mig aktiv)	LIS	0.733	0.739	0.742	—	—
12. I feel sleepy (Jag känner mig dåsig)	LIS	0.544	—	—	—	—
13. I am tense (Jag känner mig spänd)	TES	0.861	0.689	—	—	—
14. I feel relaxed (reversed) (Jag känner mig avspänd)	TES	0.793	—	—	—	—
15. I feel restless (Jag känner mig rastlös)	TES	0.446	0.463	—	—	—
16. I feel intense inner tension (Jag känner en stark inre spänning)	TES	0.709	0.902	—	—	—
17. I feel too tired to think (Jag känner mig för trött för att tänka)	CWE	0.712	0.711	0.714	—	—
18. I have difficulty concentrating (Jag har svårt att koncentrera mig)	CWE	0.787	0.786	0.789	0.773	—
19. My thinking process is slow (Jag känner mig trögtänkt)	CWE	0.852	0.853	0.850	0.860	0.902
20. I feel I am not thinking clearly (Jag kan inte tänka klart)	CWE	0.821	0.821	0.819	0.831	0.810
21. I have difficulty thinking about complex things (Det känns svårt att tänka på komplicerade saker)	CWE	0.726	0.726	0.729	0.727	0.708
22. I feel I am not focused in my thinking (Jag känner mig splittrad i tankarna)	CWE	0.545	0.545	0.542	0.544	—

Abbreviations: CWE, Cognitive Weariness; EPE, Emotional exhaustion and physiological fatigue; LIS, Listlessness; TES, Tension.

Demographic information was collected through the questionnaire included in each study, respectively. Sample characteristics are shown in Table 1.

### 3.3 Statistical Analyses

In order to examine the construct validity of the different versions of the SMBM, the dimensionality in the clinical exhaustion sample was tested by means of confirmatory factor analysis. The model fit of the five different versions of the SMBM proposed by Sundström et al. (2023) and Almén and Jansson (2021) was examined. The versions are the 4‐factor‐SMBM‐22; the modified 4‐factor‐SMBM‐19; the 3‐factor‐SMBM‐16; the 2‐factor‐SMBM‐11, and the 2‐factor‐SMBM‐6 (Almén and Jansson 2021). Table 2 shows the items included in the different versions. Model fit was examined using the following measures: *χ* ^2^/df, the Comparative Fit Index (CFI), Tucker Lewis index (TLI), Root Mean Square of Approximation (RMSEA) and Standardised Root Mean Residual (SRMR) (Hu and Bentler 1999). For CFI and TLI, values greater than 0.90 indicate an acceptable fit, and values above 0.95 a close fit. For RMSEA, values less than or equal to 0.05 are considered as good fit, values between 0.05 and 0.08 as an adequate fit, and values between 0.08 and 0.10 as a mediocre fit, whereas values above 0.10 are not acceptable. For SRMR, a rule of thumb is that values should be less than 0.05 for a good fit, whereas values smaller than 0.10 may be interpreted as acceptable (Hu and Bentler 1999; Schermelleh‐Engel, Moosbrugger, and Müller 2003). Measurement invariance tests were conducted across participant groups: patients with ED, (*n* = 149; 71.3%) versus healthy controls (*n* = 60; 28.7%) for three of the SMBM versions; SMBM‐19, SMBM‐11 and SMBM‐6. A sequential strategy was used, in which the invariance was tested at different levels. In the least restrictive model (configural), the factor structure was specified identically across groups, allowing factor loadings and intercepts to vary across groups. Second, a metric (weak) invariance model was fitted in which the factor loadings were constrained to be equal. Third, a scalar (invariant factor loadings and intercepts across groups) was fitted, and finally a strict model (invariant factor loadings, intercepts, and unique factor variances across groups). Following the guidelines proposed by Chen (2007), measurement invariance was evaluated by chi‐square difference test for nested models and changes in model fit according to CFI and RMSEA, where a change of ≥ −0.005 in CFI supplemented by a change of ≥ 0.010 in RMSEA would indicate non‐invariance (in samples < 300).

To further obtain evidence for construct validity, differences in SMBM mean scores between the exhaustion disorder sample and healthy controls were examined using independent samples *t*‐test. Effect sizes were calculated using Cohen's *d*. Cohen (1988) suggested *d* = 0.2 to be considered a small effect, 0.5 a medium effect and 0.8 a large effect. It was expected that the clinical group would score significantly higher on the SMBM compared to the healthy controls. The reliability of the different versions of SMBM was assessed using Cronbach's alpha as well as McDonald's Omega as measures of internal consistency on the clinical exhaustion disorder sample, where values of 0.70 and above indicate acceptable levels of internal consistency (Dunn, Baguley, and Brunsden 2014).

To examine criterion‐related validity, SMBM score was related to the presence of exhaustion disorder diagnosis according to the Swedish ICD‐10 (confirmed by a physician in the sample of patients). The proportion of variance of the exhaustion diagnosis variable explained by SMBM score, using logistic regression was assessed. In the regression model, SMBM score was used as independent variable and the presence of the diagnosis exhaustion disorder as dependent variable. The clinical exhaustion disorder sample and the sample of healthy controls were also used to examine the sensitivity and specificity of the different versions of the SMBM, through receiver operating characteristic (ROC) analyses. *Sensitivity* is the likelihood of an individual with the condition getting a positive test result, whereas *specificity* can be defined as the likelihood of an individual without the condition getting a negative test result. The ROC curve is a representation of the ability of the instrument to discriminate between ‘cases’ and ‘non‐cases’. Area under the curve ≥ 0.90 equals an excellent test, ≥ 0.80 good and ≥ 0.70 fair (Zou, O’Malley, and Mauri 2007). The desired cut‐off score is chosen to minimise the sum of false positive and false negative test results. The cut‐off scores presented in the result section (Table 3) were chosen based on this principle, and sensitivity and specificity for previously used cut‐offs were also reported. As mentioned in the introduction, for SMBM‐22 previous cut‐offs used are 3.75 (Grossi et al. 2003), 4.0 (Glise, Ahlborg, and Jonsdottir. 2012 2014) and 4.4 for SMBM‐22 as well as SMBM‐18 (Lundgren‐Nilsson et al. 2012). For the other versions of SMBM no cut‐offs have been established. Positive predictive values (PPV) and negative predictive values (NPV) were calculated for each cut‐off score. The PPV is interpreted as the probability of being a case at a specific cut‐off point. The PPV is indicative of the screening potential of the SMBM. The NPV is interpreted as the probability that an individual with a score below cut‐off does not have the condition.

**TABLE 3:** Sensitivity, specificity, positive predictive value (PPV) and negative predictive value (NPV) for the different versions of SMBM in detecting burnout in the clinical exhaustion disorder sample and healthy control sample. Prevalence figures related to the different cut‐off scores and versions of SMBM are provided for the general population sample and clinical primary care sample.

Cut‐off point	Sensitivity (%)	Specificity (%)	PPV (%)	NPV (%)	Prevalence in general population^a^	Prevalence in clinical primary care sample^b^
4‐Factor SMBM‐22						
3.75	98.7	88.3	95.5	96.4	22.8	85.6
4.00	96.0	90.0	96.0	90.0	18.2	81.2
4.40	89.9	95.0	97.8	79.2	11.9	66.4
4‐Factor SMBM‐19						
3.75	98.7	90.0	96.1	96.4	21.0	83.8
4.00	95.3	93.3	97.3	88.9	16.6	81.2
4.40	90.6	93.3	97.1	80.0	11.9	67.9
3‐Factor SMBM‐16						
3.75	97.3	90.0	96.1	93.1	22.9	85.2
4.00	95.3	93.3	97.3	88.9	18.8	80.8
4.40	90.6	93.3	97.1	80.0	12.5	64.6
2‐Factor SMBM‐11						
3.50	97.3	90.0	96.1	93.1	23.5	83.4
3.75	96.6	93.3	97.3	91.8	19.1	77.1
4.00	95.3	93.3	97.3	88.9	16.4	73.8
4.40	88.6	93.3	97.1	76.7	10.8	58.7
2‐Factor SMBM‐6						
3.50	96.0	93.3	97.3	90.3	22.2	80.4
3.75	94.6	95.0	97.9	87.7	17.4	71.1
4.00	91.3	95.0	97.8	81.4	15.1	66.7
4.40	83.89	95.0	97.66	70.37	9.5	53.0

^a^ Swedish general population sample (*n* = 3406).

^b^ Swedish primary‐care clinical sample (*n* = 393).

Following the recommendations proposed by Akoglu (2022) we estimated the sample size needed to reliably assess the diagnostic accuracy of the SMBM, which resulted in a minimum sample size of 114 and thus provides sufficient power in the present study.

Finally, the prevalence (%) of burnout in the three samples was calculated for the cut‐off points established by the ROC‐analyses.

The statistical analyses were performed with Jamovi 2.3.21 and JASP 0.18.3.

## 4 Results

### 4.1 Reliability and Construct Validity of the SMBM

The internal consistency for the clinical exhaustion disorder sample, was 0.94 for the 4 factor SMBM‐22. For the other versions of SMBM, internal consistencies were at similar levels (see Table 4). All values were higher than 0.80, suggesting that items are homogenous in terms of the construct they measure (Evers et al. 2013). Table 4 also displays the correlation coefficients between the different versions of the SMBM. All versions were strongly and positively correlated. There were statistically significant differences in average SMBM score between the clinical exhaustion sample and healthy controls for all versions of SMBM (see Table 5). The average score for the exhaustion disorder sample was above five points for all versions of the SMBM.

**TABLE 4:** Internal consistency (Cronbach's alpha and McDonald's omega) and bivariate correlations between the total score on the SMBM‐22, SMBM 19, SMBM‐16, SMBM‐11 and SMBM‐6 for the clinical exhaustion disorder sample (*n* = 149).

Bivariate correlations	SMBM‐22	SMBM‐19	SMBM‐16	SMBM‐11	Cronbach's alpha	McDonalds omega
SMBM‐22					0.941	0.940
SMBM‐19	0.993**				0.935	0.933
SMBM‐16	0.965**	0.972**			0.943	0.941
SMBM‐11	0.947**	0.961**	0.978**		0.914	0.911
SMBM‐6	0.897**	0.919**	0.922**	0.957**	0.849	0.844

*p < 0.05, **p < 0.01, ***p < 0.001.

**TABLE 5:** Means, standard deviations and independent samples *t*‐test of differences in SMBM score between the clinical exhaustion disorder sample (*n* = 149) and healthy controls (*n* = 60).

	Healthy control sample		Clinical exhaustion disorder sample		*T*	Cohen's *d*
	*M*	SD	*M*	SD		
SMBM‐22	2.44***	0.91	5.50	0.76	24.69	3.30
SMBM‐19	2.38***	0.92	5.50	0.77	25.06	3.35
SMBM‐16	2.38***	0.94	5.60	0.84	24.20	3.23
SMBM‐11	2.24***	0.91	5.50	0.89	23.79	3.18
SMBM‐6	2.03***	0.94	5.35	0.96	22.82	3.04

*p < 0.05, **p < 0.01, ***p < 0.001.

In order to examine the factor structure for the clinical exhaustion disorder sample, the different versions of the SMBM were subjected to confirmatory factor analysis (see Table 2 for factor loadings). Indices of model‐fit were below acceptable levels for SMBM‐22. The modified 4‐factor SMBM‐19 as well as the 2‐factor SMBM‐11 and SMBM‐6 demonstrated good model fit. The model‐fit for the 3‐factor SMBM‐16 was also good (see Table 6). In addition, measurement invariance was tested across the sample of exhaustion disorder patients and sample of healthy controls. Configural invariance for the SMBM‐6, SMBM‐11 and SMBM‐19 was supported by a good fit for the baseline model (see Table S1). Measurement invariance for the metric, scalar and strict models was not supported.

**TABLE 6:** Estimates of confirmatory factor analyses: Model‐fit indices for first‐order factor SMBM models for the clinical exhaustion disorder sample (*n* = 149).

Model	*χ*2 (df)	χ2/df	CFI	TLI	SRMR	RMSEA
4‐Factor‐SMBM‐22	403 (203)	1.99	0.875	0.857	0.074	0.081
4‐Factor‐SMBM‐19	234 (146)	1.60	0.929	0.916	0.063	0.064
3‐Factor SMBM‐16	144 (101)	1.43	0.960	0.953	0.047	0.053
2‐Factor SMBM‐11	50 (43)	1.16	0.990	0.987	0.037	0.033
2‐Factor SMBM‐6	5.88 (8)	0.74	1.00	1.01	0.025	0.001

### 4.2 Sensitivity and Specificity of the SMBM

The logistic regression showed that SMBM score accounted for a significant proportion of variance (88.5%) in the presence of exhaustion disorder diagnosis *χ* ^2^(1) = 201.23, *p* < 0.001, (Nagelkerke *r* ^2^ = 0.885). The risk of having exhaustion disorder increased 21.98 times for each additional unit on the SMBM (OR = 21.98, 95% CI = 7.36–65.65). For all versions of the SMBM, the area under the ROC curve shows that the model discriminates well between cases and non‐cases (see Table 7).

**TABLE 7:** ROC analyses of area under curve and 95% CI, for the different versions of SMBM in the clinical exhaustion disorder sample and sample of healthy controls.

	Area under curve	95% CI
SMBM‐22	0.988	0.977–0.999
SMBM‐19	0.989	0.977–1.000
SMBM‐16	0.985	0.971–1.000
SMBM‐11	0.986	0.972–0.999
SMBM‐6	0.983	0.965–1.000

Table 3 displays the sensitivity and specificity at different cut‐off scores for the different versions of the SMBM. For the SMBM‐11 and SMBM‐6 a cut‐off at 3.50 was added to the presentation as this cut‐off score provided similar levels of sensitivity and specificity as the other SMBM versions. Coordinates under the ROC curve suggest that the cut‐off with high sensitivity as well as specificity is placed around 4 (see Table 3). For the SMBM‐19, SMBM‐16 and SMBM‐11, a cut‐off at 4.00 provides a sensitivity of 95% and a specificity of 93%. For the SMBQ‐6 a cut‐off at 3.75 is associated with a similar sensitivity (95%) and specificity (95%). The positive predictive value for the cut‐off at 4.00 for SMBM‐19 shows that there is a 97.3% probability of being a case when scoring 4.00 or above. The negative predictive value for this cut‐off score implies that there is a 88.9% probability, of not being a case when scoring below 4.00. Moreover, Table 3 also shows the prevalence of exhaustion at different cut‐offs in the clinical primary care sample as well as in the sample from the general population. For example, the cut‐off 4.00 corresponds to a prevalence of exhaustion of 81.2% in the clinical primary care sample and 18.2% in the general population sample, whereas using the short version SMBM‐11 corresponds to a prevalence of 73.8% in the clinical primary care sample and 16.4% in the general population sample.

## 5 Discussion

The increase in stress‐related ill‐health calls for early detection and treatment. In this work, standardised instruments with validated clinical cut‐offs are important in order to identify persons at risk of exhaustion disorder. In this study, the reliability and construct validity of the proposed versions of the SMBM were examined in a clinical sample of patients with exhaustion disorder and criterion validity was examined by assessing sensitivity and specificity to establish clinical cut‐offs for different versions of the frequently used SMBM. To the best of our knowledge, no previous study has evaluated clinical cut‐offs for the proposed modified versions of the SMBM described above. Hence, the present study makes an important contribution to the field as the use of shorter versions of the SMBM and the SMBM‐19 would be of great utility in clinical practice as well as in research settings.

Regarding dimensionality, the findings were in line with previous studies reporting on general population samples. More specifically, the findings of Almén and Jansson (2021) and Sundström et al. (2023) showing that the SMBM‐19 has superior model‐fit to the SMBM‐22, and that the short versions SMBM‐11 and SMBM‐6 have excellent model‐fit, were corroborated by the present study. Measurement invariance was tested across the sample of exhaustion disorder patients and sample of healthy controls. Results indicated that SMBM‐6, SMBM‐11 and SMBM‐19 showed measurement invariance at the configural level, that is identical factor structure across the two groups, which means that the same indicators are measures of the constructs. However, measurement invariance at stricter levels was not supported. Furthermore, the results provided support for other aspects of construct validity as the different versions of the SMBM demonstrated strong positive inter‐correlations and as there were significant differences of large effect size in SMBM mean scores between the clinical exhaustion disorder sample and the sample of healthy controls. All versions of the SMBM displayed excellent level of internal consistency for the exhaustion disorder sample. These results are in line with findings from previous studies reporting on internal consistency and internal structure for these versions of the SMBM on general population and community samples (Sundström et al. 2023; Almén and Jansson 2021).

The ROC‐analysis on the clinical exhaustion disorder sample and sample of healthy controls demonstrated that the different versions of SMBM had excellent ability to discriminate between cases with exhaustion disorder and non‐cases. A large share of the variance in SMBM score was related to the exhaustion disorder diagnosis, which further supports the diagnostic utility of the SMBM. The ROC analysis showed that the different versions of SMBM possesses a high sensitivity and specificity. For the SMBM‐19, SMBM‐16 and SMBM‐11, a cut‐off at 4.0 was associated with the highest sensitivity (95%) and specificity (93%). This implies, that if the cut‐off is set at 4.0, 95% of the cases with exhaustion disorder and 93% of non‐cases will be correctly classified. For the brief version SMBM‐6, results indicate that 3.75 would be a suitable cut‐off as the sensitivity is higher for this cut‐off compared to a cut‐off at 4.0, but the specificity is on similar levels for both these cut‐offs. At the cut‐off 3.75, 95% of the cases and non‐cases would be correctly classified.

In relation to clinical cut‐offs previously used for the SMBM‐22, the findings from the present study indicate cut‐offs at similar levels. Our results align with those of Glise and colleagues (2012) who suggested a cut‐off at 4.0 based on clinical experience and expert consensus. Lundgren‐Nilsson et al. (2012) suggested a cut‐off at 4.4 for the 3‐factor SMBM‐18 as well as the 4‐factor SMBM‐22. Comparing the cut‐offs at 4.4 and 4.0 in our data, shows that setting the cut‐off at 4.4 is associated with a lower sensitivity and lower probability that an individual with a score below the cut‐off does not have the condition (79%), compared to a cut‐off at 4.0, for which the corresponding probability is 90%. In other words, a cut‐off set at 4.4 would result in a higher probability of false negatives compared to a cut‐off at 4.0. Based on the trade‐off between sensitivity and specificity, as well as PPV and NPV, a cut‐off of 4.0 for the SMBM‐22, SMBM‐19, SMBM‐16 and SMBM‐11 can be considered appropriate in most clinical and research contexts.

Notwithstanding, there may be situations in which using a more lenient cut‐off of 3.75 might be desirable, for example for early identification of possible cases (e.g., for preventative measures) or in contexts when it is important to ensure that people below the cut‐off indeed do not have the condition (e.g., when screening for a control group without clinical levels of burnout). A cut‐off of 4.0 applied on the clinical primary care sample in the present study results in prevalence rates of about 81% for the 22, 19 and 16 versions of SMBM, whereas a cut‐off at 4.4 would result in prevalence rates of about 65–68%. The primary care sample consisted of patients seeking primary health care for problems with stress who had not yet received a diagnosis; thus, we do not have information about the prevalence of exhaustion disorder diagnosis in this sample. Nevertheless, the relatively high prevalence of cases based on the SMBM in this sample highlights the need to complement screening instruments with clinical diagnostic interviews in health care settings.

Employing the suggested cut‐offs for SMBM on a general population sample from northern Sweden, the findings indicated a prevalence of exhaustion of 16–18% depending on which version of SMBM is used. This can be seen as an indicator that the different versions of SMBM provide reliable estimates of exhaustion, as previous studies on prevalence of exhaustion in Sweden has reported similar levels. Norlund et al. (2010) reported a prevalence of 13% for exhaustion in a random population sample. In a sample of municipal employees in northern Sweden (76% women), the prevalence of self‐reported exhaustion was 21% (Asplund et al. 2021). In a Swedish national working sample (SLOSH) the prevalence of emotional exhaustion was somewhat higher; 23.3% in 2010 (Leineweber et al. 2012). However, in that study the Maslach Burnout Inventory was used for assessing emotional exhaustion, which might explain the higher level. These findings can also be related to European figures, for which a systematic review indicated a prevalence of 18% for burnout in the Swiss working population (Al‐Gobari et al. 2022).

There are some limitations of the present study that need to be considered. Firstly, although the samples used for the two first aims comprised both patients with exhaustion disorder and healthy controls, the sample sizes were relatively small, and the distribution of SMBM scores were somewhat restricted. For the analysis of measurement invariance, a general rule of thumb for multigroup CFA is a sample size of 100 participants per group (Kline 2023), which implies that the sample of healthy controls (*n* = 60) might not provide reliable estimates of measurement invariance. Thus, the results regarding measurement invariance should be interpreted with caution and further studies of measurement invariance for SMBM across (larger) clinical and non‐clinical samples are warranted. For the ROC‐analysis however, sample size estimation showed that the study has enough power to provide reliable estimates of sensitivity and specificity as well as NPVs and PPVs. Moreover, the patients in the clinical exhaustion disorder sample were recruited from a specialised stress rehabilitation clinic, and therefore further investigation of the validity of these cut‐offs in other settings such as primary health care is needed in future studies. Concerning the general population sample, a limitation is the relatively low response rate of 40.0%, which has implications for representativeness. However, it can be noted that participation rates between 30 and 70% are at most weakly associated with bias of this kind (Galea and Tracy 2007). Similarly, 53% out of those screened for eligibility for the RECO study were included in the current study (Gavelin et al. 2018), thus the clinical exhaustion disorder sample may not be fully representative of the patient population. Worth to mention is also that the data collection for the clinical exhaustion disorder sample and general population sample was conducted in 2013. The increase of stress‐related ill‐health the past decades implies that the prevalence rates for burnout in the general population probably are even higher in today's society. Finally, while the SMBM is generally considered suitable for use across various contexts, it should be noted that one question (Item 10) specifically refers to ‘going to work’. This could present challenges for respondents who are not currently working, such as those who are on sick leave. However, despite these limitations the present study can make a significant contribution to the field in that it examines the psychometric properties of the SMBM on a clinical sample with exhaustion disorder and establishes clinical cut‐offs for the five suggested versions of the SMBM. Another important contribution of the present study is that the prevalence of exhaustion for the cut‐offs suggested are demonstrated both in a general population sample and in a primary care sample of patients experiencing elevated stress.

### 5.1 Conclusions

The current study shows that the SMBM‐19 as well as the modified versions of the SMBM ‐11 and SMBM‐6 showed good psychometric properties and favourable sensitivity (94.6%–95.3%) and specificity (93.3%–95.0%) in identifying cases with exhaustion disorder based on the cut‐off of 4.0 for the SMBM‐19, SMBM‐16 and SMBM‐11, and on the cut‐off of 3.75 for the SMBM‐6. Moreover, based on the confirmatory factor analysis on the clinical exhaustion disorder sample, we conclude that the SMBM‐19 is superior to the SMBM‐22, and thus we advocate the use of SMBM‐19 for assessing the subscales EPE, CWE, LIS and TES. Since clinical cut‐offs now are established for the SMBM‐19, SMBM‐11 And SMBM‐6 these versions can now be used as screening tools. For repeated measures in clinical as well as research settings we suggest using the brief versions SMBM‐11 or SMBM‐6. To conclude, the findings indicate that SMBM is a useful instrument for screening for stress‐related exhaustion disorder and clinical burnout.

## Author Contributions

**Anna E. Sundström:** conceptualization, methodology, formal analysis, writing initial draft, editing final version of the manuscript. **Steven Nordin:** investigation, reviewing and editing the original draft, funding acquisition. **Maria Nordin:** investigation, reviewing and editing the original draft, funding acquisition. **Anna Stigsdotter Neely:** investigation, reviewing and editing the original draft, funding acquisition. **Hanna Malmberg Gavelin:** conceptualization, methodology, investigation, data curation, writing initial draft, editing final version of the manuscript.

## Ethics Statement

The study was conducted in accordance with the Helsinki Declaration and approved by the Swedish Ethical Review Authority (Clinical exhaustion disorder sample and healthy controls; Dnr 2020‐00168; Sample from general population; Dnr 2022‐05265‐02; Clinical sample from primary health care; Dnr 2020‐00168). Participation in the study was strictly voluntary and all participants gave their informed consent.

## Conflicts of Interest

The authors declare no conflicts of interest.

## Supporting information

Table S1

## Data Availability

Data is available upon reasonable request.

## Appendix A

**TABLE A1:** Diagnostic criteria for exhaustion disorder according to the Swedish national board of health and welfare.

A. Physical and mental symptoms of exhaustion with minimum of 2 weeks duration. The symptoms have developed in response to one or more identifiable stressors which have been present for at least 6 months.
B. Markedly reduced mental energy, which is manifested by reduced initiative, lack of endurance or increase of time needed for recovery after mental efforts.
C. At least four of the following symptoms have been present most of the day, nearly every day, during the same 2‐week period: Persistent complaints of impaired memory or concentration Markedly reduced capacity to tolerate demands or to work under time pressure Emotionally instability or irritability Disturbed sleep Persistent complaints of physical weakness or fatigue Physical symptoms such as muscular pain, chest pain, palpitations, gastrointestinal problems, vertigo or increased sensitivity to sounds
D. The symptoms cause clinically significant distress or impairment in occupational, social or other important areas of functioning.
E. The symptoms are not due to the direct physiological effects of a substance (e.g. drug abuse, medication) or a general medical condition (e.g. hypothyroidism, diabetes, infectious disease).
F. If criteria for major depressive disorder, dysthymic disorder or generalised anxiety disorder are met, exhaustion disorder is set as co‐morbid condition.

*Note:* All capital letters must be fulfiled for the diagnosis.
