# Supplementary material for: Dimensionality, Sensitivity and Specificity of Different Versions of the Shirom‐Melamed Burnout Questionnaire/Measure in Clinical and Non‐Clinical Populations
Source: Stress Health. 2025 Jan 20;41(1):e70001. doi: 10.1002/smi.70001 (PMC11747081; doi:10.1002/smi.70001)
Supplement: Supplementary file 1 — Table S1 [file SMI-41-e70001-s001.docx]

**Supplementary material**

**Table 1.** Results of the multi-group tests of invariance regarding participant group (exhaustion disorder group (n=149) and healthy control group (n=60)).

|  |  |  |  |  |  |  |
| --- | --- | --- | --- | --- | --- | --- |
| Model | χ^2^ (df) | Δχ^2^(df) | CFI | ΔCFI | RMSEA | ΔRMSEA |
| **SMBM-6** |  |  |  |  |  |  |
| Configural | 19.422 (16) | – | .992 | – | .045 | – |
| Metric | 23.847 (20) | 4.425 (2) | .991 | .001 | .043 | .002 |
| Scalar | 38.814 (24) | 14.967 (4) | .966 | .025 | .077 | .034 |
| Strict | 56.442 (30) | 17.628 (6) | .938 | .053 | .092 | .015 |
| **SMBM-11** |  |  |  |  |  |  |
| Configural | 145.155 (86) | – | .942 | – | .081 | – |
| Metric | 181.439 (95) | 36.284 (9) | .916 | .026 | .093 | .012 |
| Scalar | 213.029 (104) | 31.590 (9) | .894 | .022 | .100 | .007 |
| Strict | 263.696 (115) | 50.667 (11) | .855 | .039 | .111 | .011 |
| **SMBM-19** |  |  |  |  |  |  |
| Configural | 497.024 (292) | - | .897 | - | .082 | - |
| Metric | 572.365 (307) | 75.341 (15) | .867 | .030 | .091 | .009 |
| Scalar | 612.280 (322) | 39.915 (15) | .854 | .013 | .093 | .002 |
| Strict | 682.990 (341) | 70.710 (19) | .828 | .026 | .098 | .005 |
| Note. CFI = Comparative Fit Index; RMSEA = Root Mean Square Error of Approximation; The Deltas are With Respect to the Previous Level of Measurement Invariance. | | | | | | |
